# Complete genome sequence of *‘Halanaeroarchaeum sulfurireducens’* M27-SA2, a sulfur-reducing and acetate-oxidizing haloarchaeon from the deep-sea hypersaline anoxic lake Medee

**DOI:** 10.1186/s40793-016-0155-9

**Published:** 2016-05-13

**Authors:** Enzo Messina, Dimitry Y. Sorokin, Ilya V. Kublanov, Stepan Toshchakov, Anna Lopatina, Erika Arcadi, Francesco Smedile, Gina La Spada, Violetta La Cono, Michail M. Yakimov

**Affiliations:** Institute for Coastal Marine Environment, CNR, Messina, Italy; Winogradsky Institute of Microbiology, Russian Academy of Sciences, Moscow, Russia; Department of Biotechnology, Deft University of Technology, Delft, The Netherlands; Immanuel Kant Baltic Federal University, Kaliningrad, Russia; Institute of Molecular Genetics and Gene Biology, Russian Academy of Sciences, Moscow, Russia

**Keywords:** Sulfur reduction, Strictly anaerobic, Extremely halophilic archaea, Hypersaline lake, Anoxic habitats

## Abstract

Strain M27-SA2 was isolated from the deep-sea salt-saturated anoxic lake Medee, which represents one of the most hostile extreme environments on our planet. On the basis of physiological studies and phylogenetic positioning this extremely halophilic euryarchaeon belongs to a novel genus *‘Halanaeroarchaeum’* within the family *Halobacteriaceae*. All members of this genus cultivated so far are strict anaerobes using acetate as the sole carbon and energy source and elemental sulfur as electron acceptor. Here we report the complete genome sequence of the strain M27-SA2 which is composed of a 2,129,244-bp chromosome and a 124,256-bp plasmid. This is the second complete genome sequence within the genus *Halanaeroarchaeum.* We demonstrate that genome of ‘*Halanaeroarchaeum sulfurireducens’* M27-SA2 harbors complete metabolic pathways for acetate and sulfur catabolism and for *de novo* biosynthesis of 19 amino acids. The genomic analysis also reveals that *‘Halanaeroarchaeum sulfurireducens’* M27-SA2 harbors two prophage loci and one CRISPR locus, highly similar to that of Kulunda Steppe (Altai, Russia) isolate ‘*H. sulfurireducens’* HSR2^T^. The discovery of sulfur-respiring acetate-utilizing haloarchaeon in deep-sea hypersaline anoxic lakes has certain significance for understanding the biogeochemical functioning of these harsh ecosystems, which are incompatible with life for common organisms. Moreover, isolations of *Halanaeroarchaeum* members from geographically distant salt-saturated sites of different origin suggest a high degree of evolutionary success in their adaptation to this type of extreme biotopes around the world.

## Introduction

*‘Halanaeroarchaeum sulfurireducens’* M27-SA2 was isolated from the deep-sea hypersaline anoxic lake Medee (Ionian Sea, Eastern Mediterranean, water depth 3105 m). Together with other five strains, previously isolated from shallow and terrestrial athalassic hypersaline sites of Russia and Spain [1], this haloarchaeon possesses maximum of 91–93 % 16S rDNA sequence similarity to the nearest cultured members of *Halobacteriaceae*. All *Halanaeroarchaeum* isolates represent a novel type of strictly anaerobic haloarchaea that grow best in NaCl brines close to saturation and use acetate as sole electron donor and carbon source with elemental sulfur as the only electron acceptor. Little is known about anaerobic sulfur metabolism at saturated salt conditions [2]. There is some evidence suggesting that bacterial sulfate reduction is possible under salt-saturated conditions [3], but sulfur respiration under such conditions has so far been very poorly investigated, except for the ‘*Halanaeroarchaeum’* strain HSR2^T^ and two extremely haloalkaliphilic bacteria of the order *Halanaerobiales*, *Halarsenatibacter silvermanii* and *Natroniella sulfidigena* [1, 4, 5]. Following the fact, that we were able to isolate these haloarchaea from various geographically and physico-chemically distinct hypersaline sites [1], the sulfidogenic anaerobic oxidation of acetate is likely a common feature in anoxic salt-saturated habitats, overlooked so far.

In this paper we describe the genome properties of *‘Halanaeroarchaeum sulfurireducens’* M27-SA2 providing details on carbon and sulfur metabolism, on clustered regularly interspaced short palindromic repeats (CRISPR) and on presence of prophage loci and genomic islands.

## Organism information

### Classification and features

**Fig. 1:**
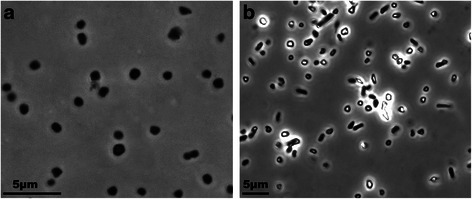
Morphology of *‘Halanaeroarchaeum sulfurireducens’* M27-SA2 cells grown on acetate (**a**) and pyruvate (**b**) as electron donors and elemental sulphur as electron acceptor. The scale bars represent 5 μm

**Fig. 2:**
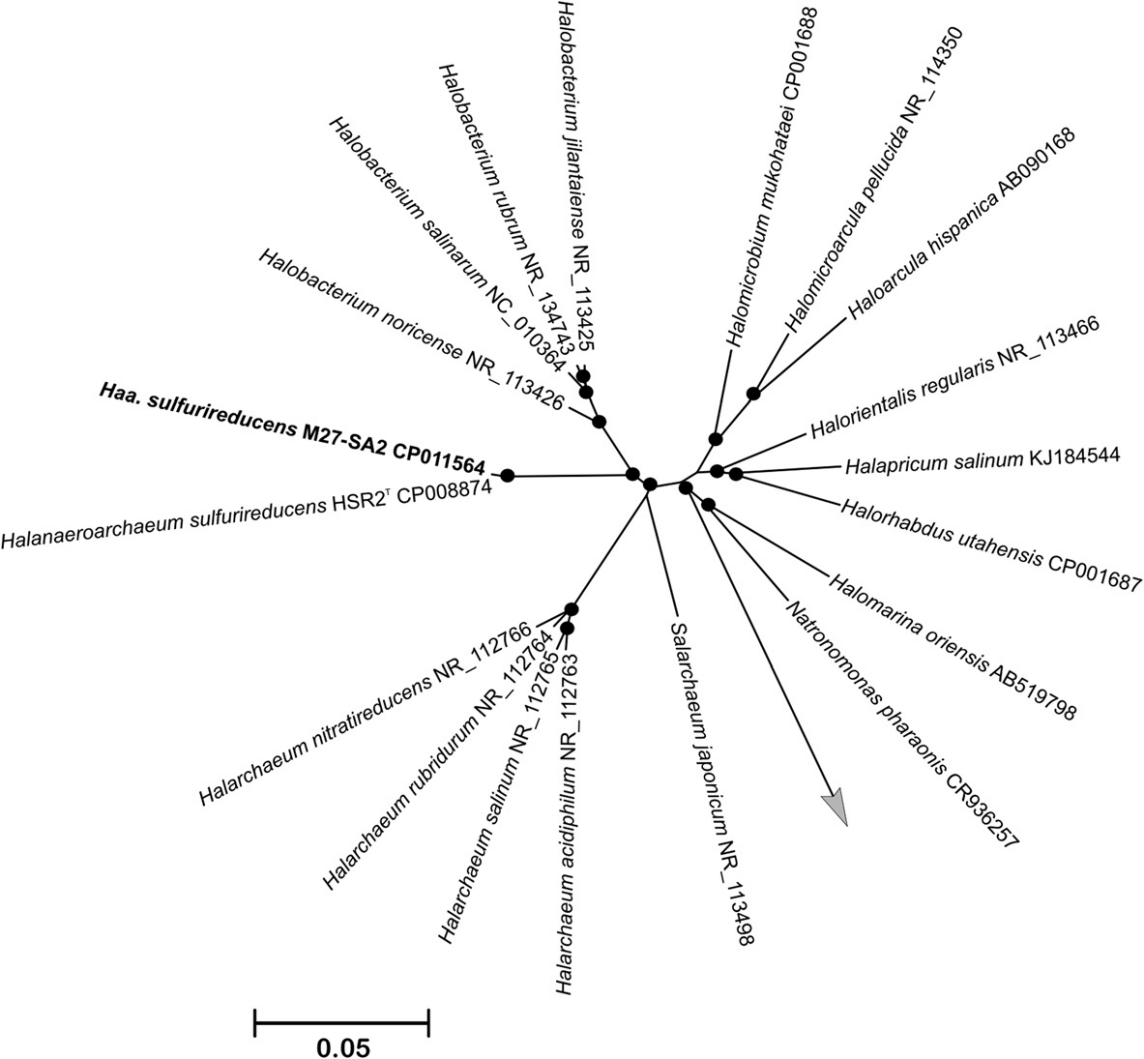
Phylogenetic tree of 16S rRNA gene sequences showing the position of *‘Halanaeroarchaeum sulfurireducens’* M27-SA2. Tree was inferred from a 16S rRNA gene sequence alignment with PAUP*4.b10 [59] using a LogDet/paralinear distance method. Support for nodes in the tree corresponds to bootstrap values for 1000 pseudo-replicates. Only bootstrap values greater than 75 % are displayed as solid circles. The tree has been arbitrarily rooted on sequence of *Natronomonas pharaonis* (D87971) and *Halomarina oriensis* (AB519798). The 16S rRNA gene sequence of *Methanohalophilus halophilus* (FN870068) was used as the outgroup. The scale bars represent a 5 % nucleotide sequence divergence

*‘Halanaeroarchaeum sulfurireducens’* M27-SA2 has typical haloarchaeal pleomorphic cell morphology, ranging from flattened rods to coccoid or irregular forms (Fig. 1). The pleomorphism of M27-SA2 strain increased with the cultivation time, as is often observed for members of the family *Halobacteriaceae*. The 16S rRNA gene of M27-SA2 exhibited 99.58 % sequence similarity with *H. sulfurireducens* strain HSR2^T^ and 97-98 % sequence similarity with clones of uncultured haloarchaea obtained from hypersaline anoxic soils, brines and sediments around the world [1] (Fig. 2).

Together with other *Halanaeroarchaeum* isolates, M27-SA2 represents the only type of obligate and strictly anaerobic haloarchaea. Most of the known cultivated extremely halophilic euryarchaeota are aerobic heterotrophs except for a few examples of facultatively anaerobic species capable of growth by fermentation [6], denitrification [7], fumarate, DMSO and TMAO reduction [8, 9]. Strain M27-SA2 was isolated from the brine (320 g l^−1^ of total salt content) of deep-sea Lake Medee (Eastern Mediterranean) collected in September 2012 at depth of 3,010 m. The collected *Medee* brine was transferred into the serum vials (120 ml) prefilled with the artificial brine to attain 230 g l^−1^ of final salinity. The artificial brine has the following composition: NaCl 200 g l^−1^; KH_2_PO_4_ 0.33 g l^−1^; yeast extract 50 mg l^−1^; Na_2_S 0.5 g l^−1^; acetate 15 mmol l^−1^; S° 2.5 g l^−1^, 10 ml l^−1^ trace elements solution (DSMZ medium 320); and 10 ml l^−1^ vitamin solution (DSMZ medium 141); pH values were adjusted to 6.7 corresponding to in situ values of the brine. Similar to all known ‘*Halanaeroarchaeum’* isolates, strain M27-SA2 grew between pH 6.7 and 8.0 (with the optimum at pH 7.2–7.5), 3.0 and 5.0 M of NaCl with the optimum growth observed at total salinity of 250 g l^−1^. Notwithstanding the isolation from the environment with permanent temperature of 15 °C [10], strain M27-SA2 has the optimal temperature of growth at 40 °C (Table 1). The isolate has a very limited metabolic profile restricted to acetate and pyruvate as the only available sources of carbon and energy and elemental sulfur as a electron acceptor [1]. Nevertheless, yeast extract should be added to the medium in concentrations of at least 10 mg l^−1^, as supplemental source of some amino acids, vitamins and cofactors which M27-SA2 likely cannot synthetize.

**Table 1:** Classification and general features of *‘Halanaeroarchaeum sulfurireducens’* M27-SA2^T^ [48]

MIGS ID	Property	Term	Evidence code^a^
	Classification	Domain *Archaea*	TAS [49]
		Phylum *Euryarchaeota*	TAS [50]
		Class *Halobacteria*	TAS [51, 52]
		Order *Halobacteriales*	TAS [53–55]
		Family *Halobacteriaceae*	TAS [56, 57]
		Genus *Halanaeroarchaeum*	TAS [1]
		Species *‘Halanaeroarchaeum sulfurireducens’*	TAS [1]
		Type strain M27-SA2^T^ (CP011564, CP011565)	TAS [1]
	Cell shape	Pleomorphic	TAS [1]
	Motility	Non-motile	TAS [1]
	Sporulation	Non-sporulating	NAS
	Temperature range	15–50 °C	TAS [1, 10]
	Optimum temperature	40 °C	TAS [1]
	pH range; Optimum	6.7–8.0; 7.2–7.5	TAS [1]
	Carbon source	Acetate, pyruvate	TAS [1]
MIGS-6	Habitat	Hypersaline anoxic lake sediments (brine)	TAS [1, 10]
MIGS-6.3	Salinity	3.0–5.0 M NaCl	TAS [1]
MIGS-22	Oxygen requirement	Strictly anaerobic	TAS [1]
MIGS-15	Biotic relationship	Free-living	TAS [1]
MIGS-14	Pathogenicity	Non-pathogen	NAS
MIGS-4	Geographic location	Lake Medee, Ionian Sea, Eastern Mediterranean	TAS [1, 10]
MIGS-5	Sample collection	24 September 2012	TAS [1, 10]
MIGS-4.1	Latitude	34°26.250N	TAS [1, 10]
MIGS-4.2	Longitude	22°19.783E	TAS [1, 10]
MIGS-4.3	Depth	3105 m	TAS [1, 10]

^a^Evidence codes – *IDA* Inferred from Direct Assay (first time in publication), *TAS* Traceable Author Statement (i.e., a direct report exists in the literature), *NAS* Non-traceable Author Statement (i.e., not directly observed for the living, isolated sample, but based on a generally accepted property for the species, or anecdotal evidence). These evidence codes are from the Gene Ontology project [58]. If the evidence is IDA, then the property was directly observed for a live isolate by one of the authors or an expert mentioned in the acknowledgements

## Genome sequencing information

### Genome project history

*‘Halanaeroarchaeum sulfurireducens’* strain M27-SA2 was selected for sequencing on the basis of its phylogenetic positions, its particular feature as a novel strictly anaerobic haloarchaeon from the deep-sea anoxic salt-saturated lake and the interest of studying this unique mechanism of anaerobic respiration, recently discovered for the first time among entire *Archaea* domain [1]. The respective genome project is deposited on the NCBI BioProject PRJNA284332 and the complete genome sequence in GenBank CP011564 and CP011565 (chromosome and plasmid) is available since 30 of September 2015. The main project information is summarized in Table 2.

**Table 2:** Project information for *‘Halanaeroarchaeum sulfurireducens’* M27-SA2

MIGS ID	Property	Term
MIGS-31	Finishing quality	Finished
MIGS-28	Libraries used	Illumina standard library, Miseq Reagent kit v2.
MIGS-29	Sequencing platforms	Illumina MiSeq System
MIGS-31.2	Fold coverage	634x chromosome, 691x plasmid
MIGS-30	Assemblers	Velvet 1.2.10, Geneious 7.1
MIGS-32	Gene calling method	Geneious 7.1, Glimmer 3.02, tRNAScan-SE
	Locus Tag	HLASA
	GenBank ID	CP011564 (chromosome)
		CP011565 (plasmid)
	GenBank date of release	30/09/2015
	BIOPROJECT	PRJNA284332
MIGS-13	Source material identifier	Isolated from the deep-sea hypersaline lake Medee, Ionian Sea, Eastern Mediterranean, water depth 3105 m. Salinity: 230 g/l; pH 6.8. Coordinates 34°26.250N, 22°19.783E.
	Project relevance	Extremophile hypersaline environments

### Growth conditions and genomic DNA preparation

Strain M27-SA2 was routinely grown anaerobically to early stationary phase in 120-ml flasks using a protocol described elsewhere [1]. Genomic DNA was isolated from the cell paste according to extraction method from Urakawa et al. [11]. DNA quality and quantity were determined with a Nanodrop spectrometer (Thermo Scientific, Wilmington, USA).

### Genome sequencing and assembly

The M27-SA2 genome was sequenced with MiSeq System technology of Illumina Inc. (San Diego, CA, USA) using paired-end 250-bp reads. The library was prepared from 1 μg of genomic DNA with NEBNext Ultra DNA library preparation kit (NewEngland Biolabs, Ipswich, USA) according to manufacturer’s instructions with insert size range of 250–750 bp and maximum of insert size distribution of 470 bp. Sequencing run resulted in 6,480,650 paired-end reads with an average read length of 250 bp, yielding 1.62 Gbp. These reads were assembled using both Velvet 1.2.10 [12] and Geneious 7.1 software. Gaps between contigs were closed with a conventional PCR-based gap closure approach and supported by manual refining with Geneious 7.1 embedded tools, resulting in a fully closed circular chromosome of 2,129,244 bp, and a circular plasmid of 124,256 bp. Together, all matching sequences provided 634× coverage for chromosome and 691× for plasmid.

### Genome annotation

Protein-coding genes were predicted by Glimmer 3.02 [13]; rRNA genes by RNAmmer 1.2 Server online tool [14]; tRNA-coding sequences by tRNAscan-SE 1.21 online tool [15]; while operon prediction was performed by the FgenesB online tool [16]. Some of structural and functional annotations were performed as it was described by Toshchakov et al. [17]. For each predicted gene similarity search was performed by Geneious 7.1 BLAST embedded tool against public amino acid sequence databases (nr, SwissProt), conserved domains families databases (Pfam, COG). Finally, annotations were manually curated using the Artemis 16.0 program [18] and refined for each gene with NCBI blastx against nr database (only for control) [19].

## Genome properties

**Fig. 3:**
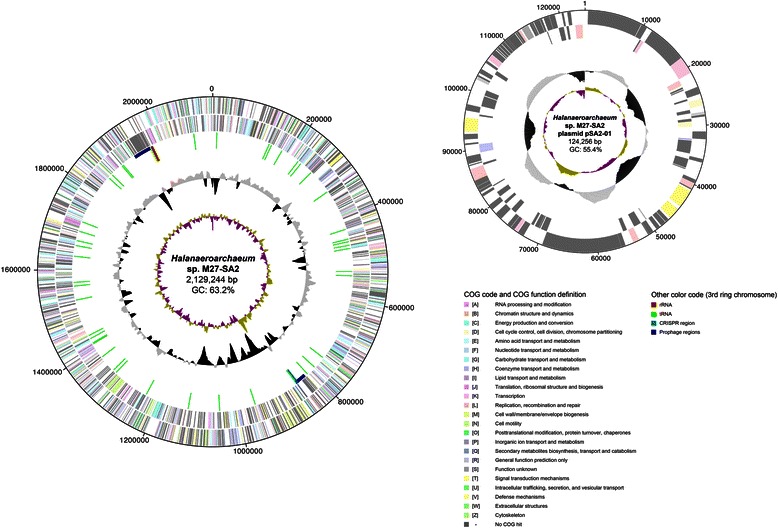
Graphical circular map of the genome of *‘Halanaeroarchaeum sulfurireducens’* M27-SA2. From outside to center: genes on forward strand (COG color-coded), genes on reverse strand (COG color-coded), RNA genes and other (only for chromosome: tRNAs green, rRNAs red, CRISPR azure, Prophages dark blue), GC content, GC skew [18, 60]

The genome of strain M27-SA2 comprises two circular replicons: a 2,129,244-bp chromosome and a 124,256-bp plasmid (Fig. 3 and Table 3). The chromosome has a 63.19 % GC content. Of the 2,200 predicted genes (88.36 % of coding density), 2,151 were protein coding genes (84.3 % started with an ATG codon, 12.9 % with a GTG, and 2.7 % with a TTG), and 49 RNAs genes (a single rRNA operon and 46 tRNAs, see Table 4). The majority of the protein-coding genes (60.72 %) were assigned with a putative function, while remaining sequences were annotated as hypothetical proteins. An assignment of genes by COGs functional categories is presented in Table 5. The plasmid has 55.39 % GC content and contains 119 protein-coding genes. Only 24 of them (20.16 %) were assigned to COGs (Table 6 and Table 7) while the remaining genes were annotated as hypothetical proteins.

**Table 3:** Genome composition for *‘Halanaeroarchaeum sulfurireducens’* M27-SA2

Label	Size (Mb)	Topology	INSDC identifier	RefSeq ID
Chromosome	2.129	circular	CP011564.1	NZ_CP011564.1
Plasmid	0.124	circular	CP011565.1	NZ_CP011565.1

**Table 4:** Chromosome statistics for *‘Halanaeroarchaeum sulfurireducens’* M27-SA2

Attribute	Value	% of total
Chromosome size (bp)	2,129,244	
DNA coding (bp)	1,860,079	87.36 %
DNA G + C (bp)	1,345,472	63.19 %
Total genes	2,200	
Protein-coding genes	2,151	97.77 %
tRNA genes	46	2.09 %
rRNA genes (5S-16S-23S)	3	0.14 %
Genes assigned to COGs	1,306	60.72 %
CRISPR repeats	1	
Average length (bp)	861	
Max length (bp)	5,619	
ATG initiation codon proteins	1,814	84.33 %
GTG initiation codon proteins	278	12.93 %
TTG initiation codon proteins	59	2.74 %

**Table 5:** Number of genes associated with the general COG functional categories for chromosome

Code	Value	% age	COG category
J	129	5.99 %	Translation, ribosomal structure and biogenesis
A	1	0.05 %	RNA processing and modification
K	79	3.67 %	Transcription
L	91	4.23 %	Replication, recombination and repair
B	3	0.14 %	Chromatin structure and dynamics
D	8	0.37 %	Cell cycle control, cell division, chromosome partitioning
V	10	0.46 %	Defense mechanisms
T	46	2.14 %	Signal transduction mechanisms
M	46	2.14 %	Cell wall/membrane/envelope biogenesis
N	28	1.30 %	Cell motility
U	9	0.42 %	Intracellular trafficking, secretion, and vesicular transport
O	58	2.70 %	Posttranslational modification, protein turnover, chaperones
C	93	4.32 %	Energy production and conversion
G	27	1.26 %	Carbohydrate transport and metabolism
E	133	6.18 %	Amino acid transport and metabolism
F	52	2.42 %	Nucleotide transport and metabolism
H	80	3.72 %	Coenzyme transport and metabolism
I	29	1.35 %	Lipid transport and metabolism
P	57	2.65 %	Inorganic ion transport and metabolism
Q	7	0.33 %	Secondary metabolites biosynthesis, transport and catabolism
R	177	8.23 %	General function prediction only
S	143	6.65 %	Function unknown
-	845	39.28 %	Not in COGs

**Table 6:** Plasmid statistics for *‘Halanaeroarchaeum sulfurireducens’* M27-SA2

Attribute	Value	% of total
Plasmid size (bp)	124,256	
DNA coding (bp)	103,887	83.61 %
DNA G + C (bp)	68,831	55.39 %
Total genes	119	
Protein-coding genes	119	
Genes assigned to COGs	24	20.16 %
Average length (bp)	873	
Max length (bp)	4,326	
ATG initiation codon proteins	78	65.55 %
GTG initiation codon proteins	28	23.53 %
TTG initiation codon proteins	13	10.92 %

**Table 7:** Number of genes associated with the general COG functional categories for plasmid

Code	Value	% age	COG category
K	4	3.36 %	Transcription
L	7	5.88 %	Replication, recombination and repair
D	2	1.68 %	Cell cycle control, cell division, chromosome partitioning
T	4	3.36 %	Signal transduction mechanisms
H	2	1.68 %	Coenzyme transport and metabolism
P	1	0.84 %	Inorganic ion transport and metabolism
R	2	1.68 %	General function prediction only
S	2	1.68 %	Function unknown
-	95	79.84 %	Not in COGs

## Insights from the genome sequence

### Genome comparisons: M27-SA2 vs HSR2 ^T^

**Fig. 4:**
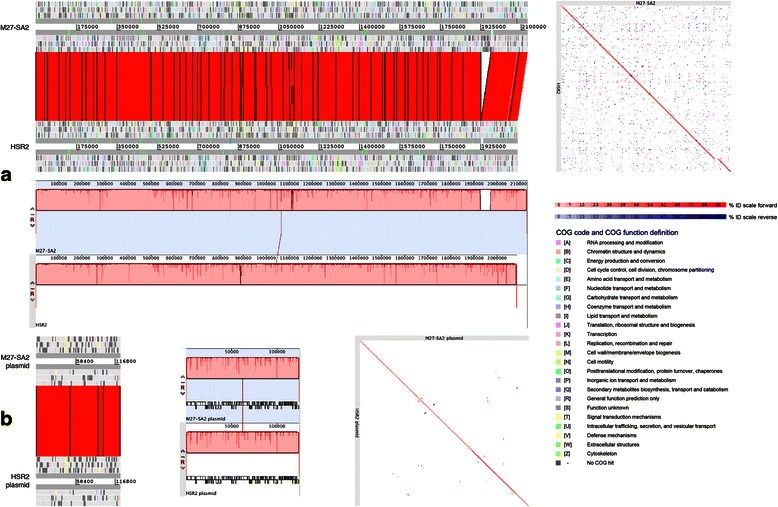
Comparison of *‘Halanaeroarchaeum sulfurireducens’* M27-SA2 vs. *‘Halanaeroarchaeum sulfurireducens’* HSR2^T^: chromosomes in (**a**) and plasmids in (**b**) ACT [20] comparisons at left (98 % ID, 40 bp minimum bitscore cutoff), LAST [21] comparisons at right (default parameters), and Mauve [22] alignments in the middle (default parameters). Genes COG color-coded, comparisons forward ID scale in red tones and reverse ID scale in blue tones

As a demonstration of their extreme similarity, the genomes of *‘Halanaeroarchaeum sulfurireducens’* M27-SA2 and *‘Halanaeroarchaeum sulfurireducens’* HSR2^T^, were compared with three different methods: the Artemis Comparison Tool program [20], the LAST web service [21], and the Multiple Genome Alignment system software (Mauve) [22]. Additionally, Double ACT web service was used to generate the required ACT comparison file. The results of these tools are shown in Fig. 4. It follows that the chromosome of M27-SA2 has high average nucleotide identities, over 97 %, to the corresponding replicon of HSR2^T^, whereas the plasmids of both isolates possess even higher average nucleotide identities values, over 99 %. The only difference with respect to the HSR2^T^ chromosome (the gap visible for all methods used) was due to the presence of an extra phage-like region (prophage 2, see Phage-like elements below). Similarly to what was found in HSR2^T^, the genome analysis of M27-SA2 identified two blocks of genes responsible for the oxidation of acetate to CO_2_ with elemental sulfur as the electron acceptor. The acetate oxidation pathway occurred by means of an ATP-dependent acetyl-CoA synthase and TCA cycle, while sulfur dissimilation could be accomplished by four different operons, coding for molybdopterin oxidoreductases (HLASA_0051-0056; HLASA_0525-0529; HLASA_0688-0694; HLASA_1275-1271). Notwithstanding, these two strains were isolated apparently from very different and geographically very distant habitats, e.g. top 10 cm-layer sediments of Kulunda Steppe (Central Russia) terrestrial hypersaline lakes (HSR2^T^), and from hypersaline brine at 3105 m depth of Lake Medee (Eastern Mediterranean) M27-SA2, we failed to find any genetic determinants reflecting such significant difference in environmental settings of these two habitats.

### Amino acid biosynthesis pathways

**Fig. 5:**
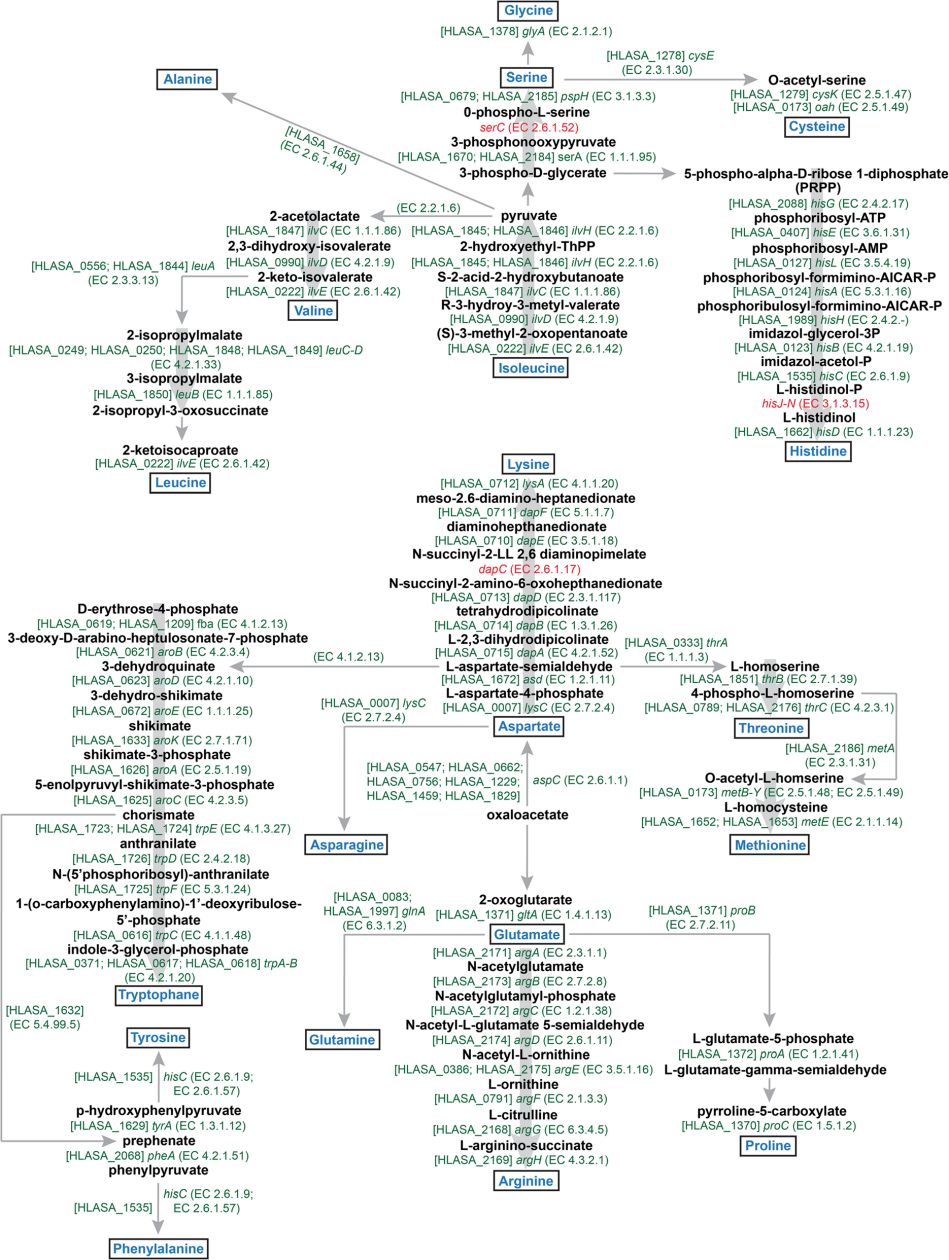
Overview of amino acid biosynthesis pathways in the genome of *‘Halanaeroarchaeum sulfurireducens’* M27-SA2. The green colour indicates the presence of a homolog coding an enzyme that may catalyse this reaction. Red colour indicates the absence of the corresponding gene in M27-SA2 genome. EC numbers are shown in parentheses, while M27-SA2 gene locus_tags are in brackets

The addition of yeast extract in amounts 10–20 mg l^−1^ is necessary for growth of strain M27-SA2, which is likely indicating that some amino acids are not synthesized or their biosynthesis could be arduous and they should be imported from the environment. We reconstructed the amino acid biosynthetic pathways of M27-SA2 using both the SEED subsystem [23] and KEGG orthology [24] assignments (Fig. 5). Similarly to what found on strain HSR2^T^ (genomes were nearly identical) this analysis indicated that the genome of strain M27-SA2 harbors all the genes required for complete synthesis of at least 19 amino acids. Seven of the eight genes involved in conversion of aspartate to lysine via tetrahydrodipicolinate, which should involve succinylated intermediates, were found. The gene *dap*C encoding N-succinyldiaminopimelate-aminotransferase was not identified in M27-SA2 genome. The pathway for the biosynthesis of isoleucine, valine, and leucine from pyruvate seems to be fully present and all genes were detected in the analyzed genome. Interestingly, a branched-chain amino acid transport system related to the permease protein Liv (HLASA_0776-0780), was detected in the M27-SA2 genome, suggesting that non-secreted amino acids could be imported via various transporters.

### CRISPR analysis

Pilercr v1.02 [25] with default parameters was used to identify Clustered Regularly Interspaced Short Palindromic Repeats array in M27-SA2 genome. The CRISPRfinder tool was used for CRISPR search as a control [26]. *Cas* genes were identified with the NCBI Blastn online tool [19]. Spacer sequences detected in CRISPR array were analyzed in order to find similarities with plasmids, phages or haloarchaeal chromosomes. Spacer sequences were blasted against nt, env_nt and wgs databases using NCBI BLAST+ blastn tool installed into web-based Galaxy platform. Additionally, spacer sequences were blasted against a local database made of several hypersaline metagenomes, including those from the anoxic hypersaline lakes *Kryos* (M. Yakimov, unpublished results) and *Thetis* [27], the hypersaline Australian lake *Tyrrell* [28], and solar salterns of *Santa Pola* [29] and *South Bay Salt* [30]. Spacers with ≤7 SNPs (80 % match or 30/37 nucleotides) were considered as positive hits. Obtained matches of at least 100 bp-long were compared to the nr and nt NCBI databases using NCBI Blast + blastx and blastn, respectively.

**Fig. 6:**
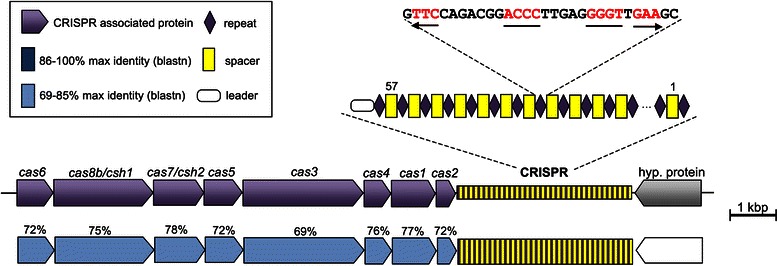
Structure of CRISPR system identified in *‘Halanaeroarchaeum sulfurireducens’* M27-SA2, with description of associated protein and repeat region found

Most sequenced so far archaeal genomes contain at least one CRISPR-Cas system [26]. DNA fragment of 13.1 kbp that included CRISPR array and associated *cas* genes was detected in the M27-SA2 genome. The CRISPR array found in M27-SA2 was practically identical to that found in *‘Halanaeroarchaeum sulfurireducens’* HSR2^T^, contained the same 30-bp direct repeat sequence (5′- GTTCCAGACGGACCCTTGAGGGGTTGAAGC -3′), and carried 57 spacers instead of 55 detected in HSR2^T^, with an average length of 37 nucleotides (individual spacer length ranged from 35 bp to 43 bp). Similarly, eight *cas* vgenes were detected in vicinity of the CRISPR array: *cas6*, *cas8b/csh1*, *cas7/csh2*, *cas5*, *cas3*, *cas4*, *cas1*, *cas2* (Fig. 6). All *cas* genes had high level of similarity to *cas* genes of *Halorhabdus tiamatea**and**Haloarcula argentinensis* (with e-value ranged from 1e^−37^ to 0.0), and thus were highly conserved between closely relative haloarchaeal genera. According to the current classification, this system was affiliated to I-B subtype or CASS7 [31].

BLASTn analysis of repeat sequence of M27-SA2 revealed several matches to haloarchaeal genomes with identical or similar (up to 3 mismatches) sequences of repeats: *Natronomonas pharaonis*DSM 2160 plasmid PL131, *Haloarcula marismortui*ATCC 43049 plasmid pNG300, *Haloarcula hispanica* N601 plasmid pHH126 and *Halorhabdus tiamatea* SARL4B.

Spacers extracted from *‘Halanaeroarchaeum sulfurireducens’* M27-SA2 were identical to that found in HSR2^T^, although two spacers (#36 and #37) were found only in M27-SA2.

When we compared 57 spacers extracted from *‘Halanaeroarchaeum sulfurireducens’* M27-SA2 to nt, env_nt and wgs Genbank databases, no matches were obtained. We blasted the spacers against metagenomic sequences of samples obtained from aforementioned hypersaline lake environments. This analysis identified six spacers that matched the metagenomic sequences (protospacers) obtained from the salt-saturated lakes *Kryos* and *Tyrrel* (Table 8). Spacer #37 matched to DNA fragment from lake Kryos metagenome that contained CRISPR array with repeat type of *H. marismortui*ATCC 43049 plasmid pNG400. These results suggest the occurrence of bacteria or/and viruses transfer between hypersaline biotopes. The spacer #7 matched to metagenomic sequences from viral fraction of lake *Tyrrell*. When those reads were compared to nt database, hits to viral contigs eHP-1, eHP-4, eHP-15 and eHP-19 from *Santa-Pola* solar saltern [29], and to the sequence of uncultured virus clone from Tunisian solar salterns [32] were found. Four other spacers (#24, #34, #52 and #54) matched to sequences from viral fraction of metagenome from lake *Tyrrell*. No homology in the GenBank was found. These results demonstrate that *‘Halanaeroarchaeum sulfurireducens’* M27-SA2 CRISPR spacers, likewise HSR2^T^ CRISPR spacers, target mobile genetic elements that have been identified in distant solar and deep-sea hypersaline lakes, suggesting that this haloarchaeon could have been adapted to yet unexplored haloviruses.

**Table 8:** CRISPR spacers analysis in the chromosome of *‘Halanaeroarchaeum sulfurireducens’* M27-SA2

Spacer #	Match to metagenomic library	# of mismatches	5′ PAM sequence	Match to GenBank nt database
7	Lake *Tyrrell* (SRR402046)	4	TTT	gb|JQ807236.1|, environmental Halophage eHP-15
24	Lake *Tyrrell* (SRR402045)	3	CTC/TGC	no
34	Lake *Tyrrell* (SRR402046)	2	TTC/TTT	no
37	Lake *Kryos* (unpublished)	0		gb|AY596293.1|, *Haloarcula marismortui* ATCC 43049 plasmid pNG400, CRISPR array
52	Lake *Tyrrell* (SRR402046)	5	GTG	no
54	Lake *Tyrrell* (SRR402045)	5	TTC	no

The presence of a short protospacer adjacent motif located upstream of the protospacer is required for immunity of type I CRISPR-Cas system [33]. The PAM sequence varies in different CRISPR subsystems. It has been shown that archaeal I-B CRISPR-Cas system has several different PAM sequences upstream of the protospacer: TTC, ACT, TAA, TAT, TAG and CAC for *Haloferax**volcani* [34], TTC for *Haloquadratum walsbyi* [34], TTT, TTC, TTG, and CCC for *H. hispanica* [35]. Our analysis detected several variants of trinucleotide sequence upstream of different protospacers (Table 8). Interestingly, most of them had TTC/TTT sequence, which are highly conserved among archaeal I-B subsystems reported so far [36]. This fact suggests that CRISPR-Cas system is likely active in *‘Halanaeroarchaeum sulfurireducens’* M27-SA2.

### Phage-like elements

**Fig. 7:**
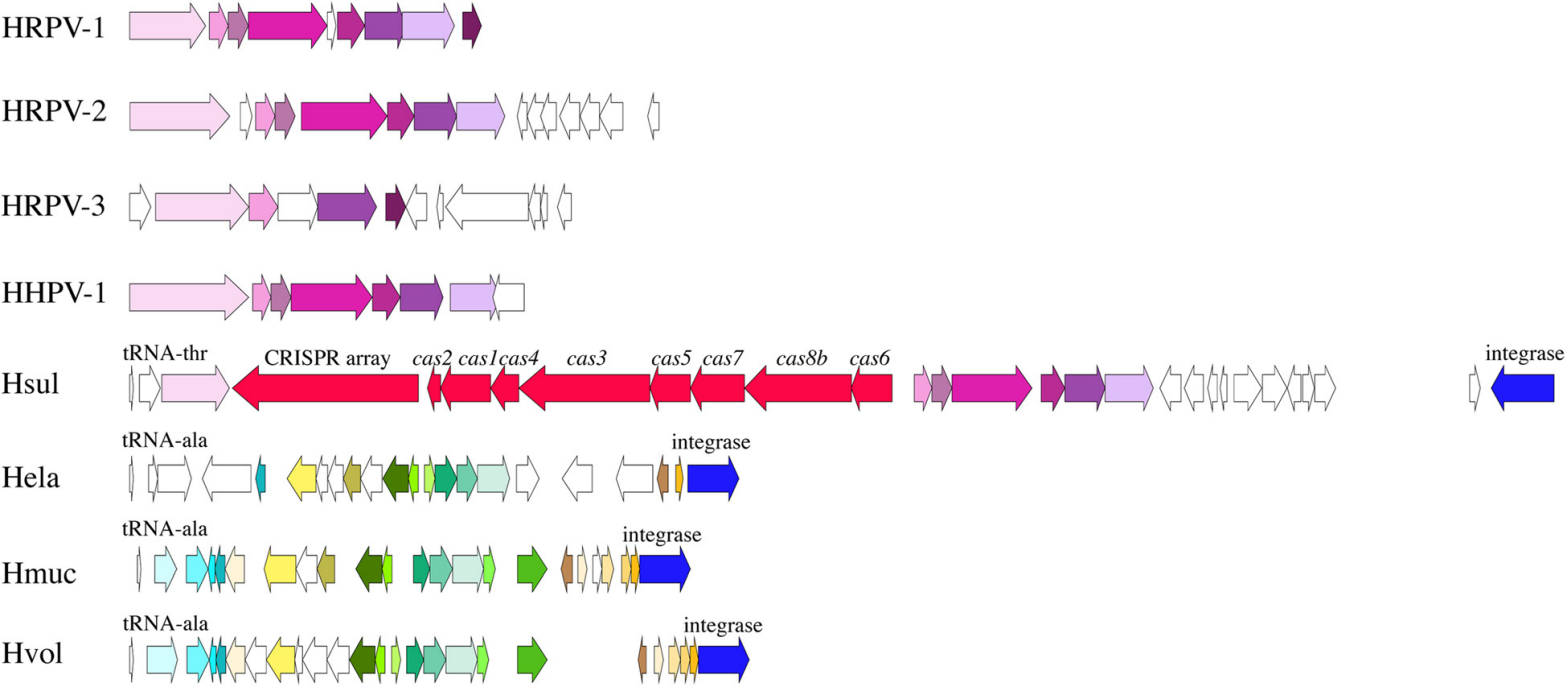
Comparative genome map with genome alignment results. Relative lytic pleomorphic viruses HRPV1, HRPV2, HRPV3, HHPV1 have seven core genes (colored in shades of magenta) that are shared between them and a prophage 1 from *‘Halanaeroarchaeum sulfurireducens’* M27-SA2. Relative prophages of *Haloferax* Hela, Hmuc, Hvol (coloured in shades of green) have tRNA and XerC/D integrase/recombinase flanking their genomes. The same flanks has the genome of *‘Halanaeroarchaeum sulfurireducens’* M27-SA2 prophage Hsul. CRISPR array and cas genes are colored in red. XerC/D integrase/recombinase is colored in blue. The same colors of the genes in genomes represent homologous genes. Short designations used: HRPV1, *Halorubrum* pleomorphic virus 1; HRPV2, *Halorubrum* pleomorphic virus 2; HRPV3, *Halorubrum* pleomorphic virus 3; HHPV1, *Haloarcula* pleomorphic virus 1; Hsul, *‘Halanaeroarchaeum sulfurireducens’* M27-SA2 prophage 1; Hela; *Haloferax elongans* contig AOLK01000011 [38]; Hmuc, *Haloferax mucosum* contig AOLN01000011 [42]; Hvol, *Haloferax volcanii* chrom1 [42]

It has been estimated that 60–70 % of prokaryotic genomes deposited to GenBank contain prophage sequences [37]. We analyzed the genome of *‘Halanaeroarchaeum sulfurireducens’* M27-SA2 in terms of presence of prophages. Apparently, there were two fragments of 28.5 kbp (prophage 1) and 49.5 kbp (prophage 2) that contained clusters of genes of viral origin. Manual annotation of prophage 1 gave best matches to putative ORFs of haloarchaeal pleomorphic phages HRPV1, HRPV2, HRPV3, HRPV6, HHPV1 and HHPV2 of *Haloferax lucentense**,* pHK2 plasmid, and prophages in the genomes of *Halomicrobium mukohataei* and *Haloferax volcanii*. The genome of prophage 1, presents in both M27-SA2 and HSR2^T^ strains, was located near the tRNA gene, a common site for prophage insertions [38], and contained a putative XerC/D integrase/recombinase gene on the opposite to tRNA gene flank. Presence of an integrase and a tRNA insertion site could be interpreted as indicator of an active prophage. The alignment of the genome of prophage 1 and its closest relatives shows that prophage 1 contains ORFs homologous to the whole set of core genes in the genomes of lytic pleoviruses HRPV-1, HRPV-2, HRPV-3 and HHPV-1 (Fig. 7 and Tables 9 and 10). However, we have found a single CRISPR array and eight associated cas genes of I-B subtype inside the genome of prophage 1. The insert is ~14 kbp long and occupies half of the prophage 1 genome (~28.5 kbp). Earlier, the entire CRISPR-Cas system including cas genes of I-F type and a CRISPR array was found in the genome of myovirus ICP1 of *Vibrio**cholera* serogroup O1 [39]. Most of the spacers from ICP1 CRISPR array targeted PICI-like element from the genome of *V. cholera*, an excised circular DNA fragment, which becomes induced and interferes with phage reproduction during infection. Therefore, bacteriophages can acquire CRISPR-Cas systems from the host genome or from the environment through natural transformation of the host cell and use it to abolish anti-phage cellular mechanisms. Two opposite suggestions could be made based on the presence of the CRISPR-Cas system in the prophage 1. On one hand, the insert of DNA fragment containing CRISPR-Cas system equal to the length of the “viral” fragment of the prophage 1 as well as presence of a number of small ORFs of host origin interspaced by long non-coding regions with numerous stop codons on the right wings of its genome (see Fig. 7) would compromise release of viral particles. On the other hand, the pleomorphic nature of the prophage 1 could allow of formation viable particles with extended genomes, as viral packaging and release are driven by a budding vesicle from the plasma membrane, and the size of the genome therefore dictates the size of the vesicle [40]. According to this logic, CRISPR-Cas system of prophage 1 would not be active during lysogenic stage of infection as the expression of most of the lytic genes of a prophage are usually shut off [41], but could become active during lytic stage of infection and be used to overcome bacterial defense. Additional experiments can be designed and performed to distinguish between these two scenarios.

**Table 9:** NCBI blastp results for prophage 1 region of *‘Halanaeroarchaeum sulfurireducens’* M27-SA2

locus_tag	Length (bp)	NCBI blastp best hit	e-value	gb accession
HLASA_0843	1239	integrase [*Halorhabdus utahensis*]	6.00e-176	WP_015789480.1
HLASA_0844	213	hypothetical protein [*Halomicrobium katesii*]	5.00e-05	WP_018258875.1
HLASA_0845	417	DNA-binding protein [*Halostagnicola larsenii* XH-48]	1.00e-79	AHG02386.1
HLASA_0846	231	hypothetical protein [*Salinarchaeum* sp. Harcht-Bsk1]	5.00e-37	WP_020446487.1
HLASA_0847	267	hypothetical protein [uncultured archaeon A07HR60]	6.00e-07	WP_023506388.1
HLASA_0848	489	hypothetical protein [*Halarchaeum acidiphilum*]	4.00e-94	WP_020221057.1
HLASA_0849	552	hypothetical protein [*Halarchaeum acidiphilum*]	9.00e-119	WP_044957257.1
HLASA_0850	141	MULTISPECIES: hypothetical protein [*Haloarcula*]	2.00e-21	WP_004594507.1
HLASA_0851	189	hypothetical protein [*Haloquadratum walsbyi*]	4.00e-33	WP_021056874.1
HLASA_0852	381	hypothetical protein [*Halorubrum* sp. T3]	2.00e-82	WP_026046123.1
HLASA_0853	423	hypothetical protein [*Halorubrum saccharovorum*]	3.00e-44	WP_004048594.1
HLASA_0854	954	hypothetical protein [*Halomicrobium mukohataei*]	6.00e-27	WP_015761810.1
HLASA_0855	798	hypothetical protein HRPV-1_gp7 [*Halorubrum* pleomorphic virus 1]	2.00e-26	YP_002791892.1
HLASA_0856	474	unknown [*Haloarcula hispanica* pleomorphic virus 1]	6.00e-19	YP_003411999.1
HLASA_0857	1581	MULTISPECIES: hypothetical protein [*Haloferax*]	8.00e-52	WP_008576942.1
HLASA_0858	399	gp3 [*Haloarcula hispanica* pleomorphic virus 2]	6.00e-47	YP_009008689.1
HLASA_0859	351	hypothetical protein [*Haloarcula argentinensis*]	5.00e-38	WP_005538312.1
HLASA_0860	813	CRISPR-associated protein Cas6 [*Halorhabdus tiamatea*]	7.00e-151	WP_008524857.1
HLASA_0861	2112	CRISPR-associated protein Csh1 [*Halorhabdus tiamatea*]	0	WP_008524855.1
HLASA_0862	1068	CRISPR-associated protein, Csh2 family [*Natronorubrum sulfidifaciens*]	0	WP_008164963.1
HLASA_0863	801	CRISPR-associated protein Cas5 [*Halorhabdus tiamatea*]	2.00e-124	WP_008524852.1
HLASA_0864	2589	CRISPR-associated helicase, Cas3 [*Halorhabdus tiamatea*]	0	WP_020936219.1
HLASA_0865	555	CRISPR-associated protein Cas4 [*Halorhabdus utahensis*]	4.00e-99	WP_015789200.1
HLASA_0866	993	CRISPR-associated protein Cas1 [*Halorhabdus utahensis*]	0	WP_015789199.1
HLASA_0867	264	CRISPR-associated endonuclease Cas2 [*Halorhabdus utahensis*]	1.00e-37	WP_015789198.1
repeat_region	3798	CRISPR repeat region		
HLASA_0868	1338	replication-related protein [*Natrinema versiforme*]	5.00e-145	WP_006432249.1
HLASA_0869	411	PREDICTED: multidrug and toxin extrusion protein 1 [*Cavia porcellus*]	0.1	XP_003465357.1

**Table 10:** NCBI blastp results for prophage 2 region of *‘Halanaeroarchaeum sulfurireducens’* M27-SA2

locus_tag	Length (bp)	NCBI blastp best hit	e-value	gb accession
HLASA_2001	1347	integrase [*Natronobacterium gregoryi*]	3.00e-178	WP_005577816.1
HLASA_2002	186	no		
HLASA_2003	1125	hypothetical protein [*Haloferax* sp. ATB1]	5.00e-11	WP_042662540.1
HLASA_2004	996	zinc finger SWIM domain protein [*Haloarcula vallismortis*]	5.00e-14	WP_004517574.1
HLASA_2005	219	hypothetical protein [*Halosimplex carlsbadense*]	4.00e-15	WP_006885565.1
HLASA_2006	1071	ORC / cell division control protein 6 [*Haloarcula amylolytica*]	2.00e-98	WP_008307569.1
HLASA_2007	660	hypothetical protein [*Haloarcula amylolytica*]	9.00e-59	WP_008312971.1
HLASA_2008	978	hypothetical protein [*Haloarcula amylolytica*]	3.00e-144	WP_008312969.1
HLASA_2009	639	PHP domain-containing protein [*Haloarcula amylolytica*]	3.00e-106	WP_008312967.1
HLASA_2010	912	decaprenyl-phosphate phosphoribosyltransferase [*Haloarcula amylolytica*]	0	WP_008312966.1
HLASA_2011	423	hypothetical protein [*Haloarcula vallismortis*]	2.00e-29	WP_004518340.1
HLASA_2012	1092	NAD-dependent epimerase/dehydratase [*Haloarcula amylolytica*]	0	WP_008312962.1
HLASA_2013	1299	hypothetical protein [*Anaerolinea thermophila*]	2.00e-35	WP_013559525.1
HLASA_2014	810	concanavalin A-like lectin/glucanases family protein [*Halorubrum* sp. AJ67]	2.00e-18	CDK38289.1
HLASA_2015	597	hypothetical protein OSG_eHP34_00135 [Environmental halophage eHP-34]	1.00e-28	AFH22760.1
HLASA_2016	576	hypothetical protein HGTV1_28 [halovirus HGTV-1]	2.00e-11	YP_008059236.1
HLASA_2017	912	hypothetical protein PhiCh1p32 [*Natrialba* phage PhiCh1]	2.00e-29	NP_665949.1
HLASA_2018	1263	baseplate J protein [haloarchaeon 3A1_DGR]	2.00e-178	WP_039401004.1
HLASA_2019	363	hypothetical protein [haloarchaeon 3A1_DGR]	7.00e-15	WP_021074727.1
HLASA_2020	681	hypothetical protein [*Natrialba magadii*]	3.00e-34	WP_004268274.1
HLASA_2021	879	hypothetical protein [haloarchaeon 3A1_DGR]	4.00e-97	WP_021074730.1
HLASA_2022	345	hypothetical protein [haloarchaeon 3A1_DGR]	3.00e-43	WP_039401001.1
HLASA_2023	564	hypothetical protein [haloarchaeon 3A1_DGR]	1.00e-35	WP_021075289.1
HLASA_2024	3123	prophage pi3 protein 14 [*Halalkalicoccus jeotgali*]	1.00e-55	WP_008414607.1
HLASA_2025	477	hypothetical protein [haloarchaeon 3A1_DGR]	1.00e-13	WP_021074542.1
HLASA_2026	1296	hypothetical protein [haloarchaeon 3A1_DGR]	2.00e-155	WP_039400994.1
HLASA_2027	588	hypothetical protein [*Natrialba magadii*]	1.00e-37	WP_004268261.1
HLASA_2028	447	hypothetical protein [haloarchaeon 3A1_DGR]	1.00e-45	WP_039400992.1
HLASA_2029	285	hypothetical protein [haloarchaeon 3A1_DGR]	6.00e-29	WP_021074547.1
HLASA_2030	387	hypothetical protein EL22_16975 [*Halostagnicola* sp. A56]	2.00e-05	KDE59819.1
HLASA_2031	366	hypothetical protein HHTV1_22 [halovirus HHTV-1]	2.00e-05	YP_008058712.1
HLASA_2032	1140	major capsid protein go21 [halovirus HHTV-1]	3.00e-91	YP_008058711.1
HLASA_2033	453	acyl dehydratase [*Haloferax mediterranei*]	0.032	WP_014732690.1
HLASA_2034	1221	PREDICTED: myosin-9-like [*Nelumbo nucifera Gaertn.*]	2.00e-04	XP_010274858.1
HLASA_2035	294	no		
HLASA_2036	216	no		
HLASA_2037	417	no		
HLASA_2038	339	hypothetical protein [*Natronobacterium gregoryi*]	5.00e-09	WP_005577927.1
HLASA_2039	669	hypothetical protein [*Haloterrigena thermotolerans*]	9.00e-23	WP_006649646.1
HLASA_2040	381	hypothetical protein [*Halovivax ruber*]	2.00e-08	WP_015300135.1
HLASA_2041	1605	uncharacterized protein BN903_58 [*Halorubrum* sp. AJ67]	1.00e-48	CDK39659.1
HLASA_2042	1710	hypothetical protein HALG_00007 [*Halorubrum* phage CGphi46]	1.00e-131	YP_008126542.1
HLASA_2043	444	hypothetical protein HCTV2_15 [halovirus HCTV-2]	4.00e-29	YP_008058377.1
HLASA_2044	1320	DNA methylase [*Halostagnicola* sp. A56]	4.00e-120	KDE56926.1
HLASA_2045	219	hypothetical protein OSG_eHP14_00030 [Environmental halophage eHP-14]	4.00e-08	AFH21986.1
HLASA_2046	171	hypothetical protein [*Haloarcula argentinensis*]	8.00e-05	WP_005538080.1
HLASA_2047	303	no		
HLASA_2048	3660	hypothetical protein [*Natrialba magadii*]	0	WP_004217537.1

Another prophage 2 is ~43.5 kbp in length and containes 49 ORFs. One of the ORFs encodes a putative tape tail measure protein, which is a key feature of *Siphoviridae* morphological family. The closest homologues of ORFs of prophage 2 were related to several haloviruses: Bj1 (siphovirus that infects *Halorubrum*), phiCh1 (myovirus of *Natrialba*), HHTV1 (siphovirus of *H. hispanica*), HGTV1 (myovirus of *Halogranum* sp.), prophages in the *Haloferax mucosum* and *Haloferax elongans* genomes [42] and to environmental viral contigs (environmental halophages eHP-2, eHP-14, eHP-32, eHP-34, eHP-36). Interestingly, prophage 2 encodes an adenine-specific DNA methylase, which could be responsible for protection from host restriction endonucleases through methylation of the phage genome [43]. Prophage 2 is located next to a tRNA integration site and has a XerC/D integrase/recombinase gene on the opposite side of the genome: both features are always present as a part of a viable prophage. Archaeal tailed viral genomes integrated into cellular chromosomes have been identified before [44]. The fact that the closest host homologue in the database (*H. sulfurireducens* strain HSR2^T^, accession number CP008874) doesn’t contain a sequence of the prophage 2 suggests recent acquisition of this prophage. Viruses of *Euryarchaeota* encompass different morphologies, including spindle-shaped viruses (*Salteproviridae*), pleomorphic viruses (*Pleolipoviridae*), head-tailed viruses (*Caudovirales*), spherical viruses (*Plasmaviridae*), and unclassified icosahedral dsDNA viruses with an inner lipid layer [45]. Therefore, the presence of two different prophages in the same genome of *H. sulfurireducens* M27-SA2 suggests that archaea in hypersaline environemnts located at the bottom of the Mediterranean Sea are exposed to a constant threat of phage predation.

### Genomic islands

**Fig. 8:**
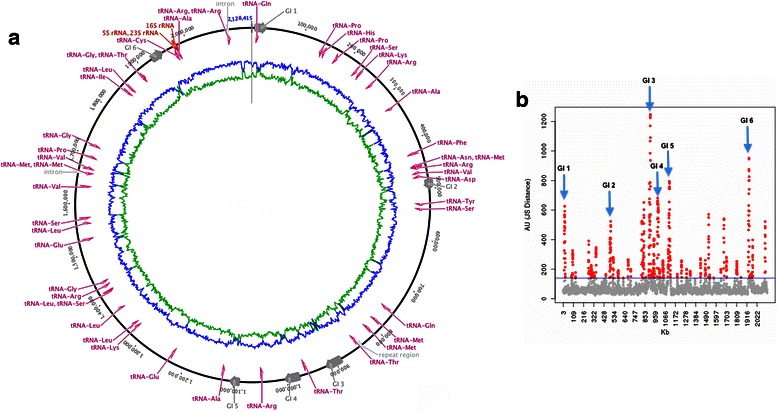
Localization of GIs on the chromosome of *‘Halanaeroarchaeum sulfurireducens’* M27-SA2, as predicted by SWGIS [46] **a** (*grey arrows* on circular map) and GOTHAM [47] **b** (red dots). Common predicted regions of both methods are highlighted in **b**) (blue arrows)

Horizontally transferred genomic islands (GIs) in *‘Halanaeroarchaeum sulfurireducens’* M27-SA2 genome were determined by the SeqWord Gene Island Sniffer program [46] and by the GOHTAM online tool [47]. The results of both GI identification methods are shown in Fig. 8. Six putative GIs characterized by alternative oligonucleotide usage patterns were detected by SWGIS, while GOHTAM search returned many short region (overall 52, see Fig. 8b) in addition to the six previously identified, probably due to a lower default sensitivity threshold of the latter method. Predicted GIs harbored mainly hypothetical proteins, transposases, glycosyltransferases (many in the third GI), and other enzyme-coding genes (transport and metabolism), a tRNA in the first GI, while the sixth GI covers the prophage 2 region (see above), not present in *‘Halanaeroarchaeum sulfurireducens’* HSR2^T^.

## Conclusions

In this manuscript we report on the complete genome sequence of *‘Halanaeroarchaeum sulfurireducens’* M27-SA2 which is composed of a 2,129,244-bp chromosome and a 124,256-bp plasmid. This is the first indication of the presence of obligate anaerobic sulfur-respiring haloarchaeon in deep-sea hypersaline anoxic lakes located on the seabed of Eastern Mediterranean Sea. This finding has significance for understanding of the biogeochemical functioning of these harsh ecosystems. Genome comparisons, analisys of amino acid biosynthesis pathways, CRISPR, phage-like elements and genomic islands was performed to understand the evolutionary success of *Halanaeroarchaeum* members in their adaptation to extreme biotopes around the world.
